# Modification of Mcl-1 alternative splicing induces apoptosis and suppresses tumor proliferation in gastric cancer

**DOI:** 10.18632/aging.103766

**Published:** 2020-10-14

**Authors:** Yonghong Li, Xiaoling Gao, Chaojun Wei, Rui Guo, Hui Xu, Zhongtian Bai, Jianye Zhou, Jun Zhu, Wanxia Wang, Yu Wu, Jingzhe Li, Zhongliang Zhang, Xiaodong Xie

**Affiliations:** 1 Key Laboratory of Preclinical Study for New Drug of Gansu Province, School of Basic Medical Sciences, Lanzhou University, Lanzhou 730000, China; 2 NHC Key Laboratory of Diagnosis and Therapy of Gastrointestinal Tumor, Gansu Provincial Hospital, Lanzhou 730000, China; 3 The Second Department of General Surgery, Lanzhou University First Hospital, Lanzhou 730000, China; 4 Key Lab of Stomatology of State Ethnic Affairs Commission, Northwest Minzu University, Lanzhou 730030, China; 5 Pathology Department, Lanzhou University First Hospital, Lanzhou 730000, China; 6 Oncology Department, The First Hospital of Lanzhou, Lanzhou 730050, China

**Keywords:** Mcl-1, splicing, apoptosis, gastric cancer, therapy

## Abstract

Splicing dysregulation, which leads to apoptosis resistance, has been recognized as a major hallmark for tumorigenesis and cancer progression. Targeting alternative splicing by either increasing pro-apoptotic proteins or inhibiting anti-apoptotic proteins in tumor cells may be an effective approach for gastric cancer (GC) therapy. However, the role of modulation of alternative splicing in GC remains poorly understood. In this study, to the best of our knowledge, the unbalanced expression of the myeloid cell leukemia-1 (Mcl-1) splicing variants, Mcl-1L and Mcl-1S, was identified in GC patients for the first time. Increasing anti-apoptotic Mcl-1L and decreasing pro-apoptotic Mcl-1S expression levels were correlated with tumor proliferation and poor survival. *In vitro* data showed that a shift in splicing from Mcl-1L to Mcl-1S induced by treatment with Mcl-1-specific steric-blocking oligonucleotides (SBOs) efficiently decreased Mcl-1L expression, increased Mcl-1S expression, and accelerated tumor cell apoptosis in a dose-dependent manner. Additionally, mouse xenotransplant models confirmed that modification of Mcl-1 alternative splicing increased tumor cell death and suppressed tumor proliferation. This study demonstrated that the modification of Mcl-1 splicing might stimulate the pro-apoptotic factor and inhibit the anti-apoptotic protein to induce significant apoptosis. Thus, this finding provided a strategy for cancer therapy, according to which SBOs could be used to change the Mcl-1 splicing pattern, thereby inducing apoptosis.

## INTRODUCTION

Apoptosis, an evolutionarily conserved process determined by apoptotic protein expression, is essential for tumor cell elimination and cancer suppression [1–3]. An apoptosis-resistant state is commonly seen in the initiation, progression, and treatment failure stages of human cancer [4–6], and molecular inhibitors that target anti-apoptotic proteins have been increasingly developed over the past three decades. Drugs that target the anti-apoptotic protein Bcl-2 have emerged as “breakthrough therapies,” and have been approved for chronic lymphocytic leukemia patients. However, the efficacy of most inhibitors that target anti-apoptotic proteins is unsatisfactory in clinical application, especially in solid tumors [7–10], and there is a concern that frequently spliced variants of apoptotic proteins diminish the ability of drugs to bind tightly to their targets, consequently limiting their efficacy [11–13]. This has ignited interest in developing new strategies that target alternative splicing to regulate tumor cell apoptosis [14, 15].

Alternative splicing, a vast source of biological regulation, occurs in nearly all types of human precursor messenger RNA (pre-mRNA) and plays a decisive role in producing protein diversity and controlling cell growth and development [16, 17]. Accumulating evidence has demonstrated that many apoptosis-related genes are subjected to alternative splicing, resulting in subtly different isoforms with antagonistic (anti- or pro-apoptotic) functions [18, 19]. Moreover, the unbalanced anti- and pro-apoptotic isoform expression results from alternative splicing of key apoptotic factors, such as Bcl-x, Bcl-2L11, and myeloid cell leukemia-1 (Mcl-1), possibly promoting cancer initiation and/or maintenance [20–22]. Thus, the shift from an anti-apoptotic isoform to a pro-apoptotic isoform, induced by regulation of alternative splicing, may not only surmount the effects of the anti-apoptotic isoform but also promote the benefits of the pro-apoptotic isoform. Thus, this is a promising strategy for facilitating tumor cell death and restraining cancer [15, 23].

However, the alternative splicing pattern of apoptotic factors in gastric cancer (GC) remains poorly understood, and regulation of apoptosis by targeting alternative splicing in GC therapy remains unexplored. Mcl-1, an important member of the Bcl-2 gene family, is traditionally regarded as an anti-apoptotic factor. Mcl-1 pre-mRNA undergoes alternative splicing, producing anti-apoptotic Mcl-1L and pro-apoptotic Mcl-1S isoforms [24–26]. Additionally, Mcl-1L over-expression and Mcl-1S under-expression have recently emerged as key survival and resistance factors involved in the evasion of apoptosis in some solid tumors [22, 27, 28]. Moreover, Mcl-1L short hairpin RNA knockdown reduces oral cancer cell viability and growth, and the shift in the pre-mRNA splicing pattern from Mcl-1L to Mcl-1S can dramatically enhance apoptosis in basal cell carcinoma and non-small cell lung cancer [22, 29, 30]. These studies have demonstrated that the unbalanced isoform expression is involved in tumor development; therefore, modulation of Mcl-1 splicing may promote apoptosis and suppress tumor development. However, Mcl-1 splicing patterns in GC remain unexplored, and there is little data available on the regulation of apoptosis by targeting Mcl-1 alternative splicing for GC treatment.

This study focused on Mcl-1 to explore a new anti-GC strategy, which involved targeting alternative splicing of apoptotic factors. For the first time, based on clinical research, we demonstrated that prominent Mcl-1L and reduced Mcl-1S expression levels were closely correlated with GC development. Next, we systematically verified that the shift in the Mcl-1 splicing pattern from Mcl-1L to Mcl-1S induced significant apoptosis, consequently suppressing tumor viability and proliferation *in vitro* and *in vivo*. Therefore, our study could contribute to the development of a new therapeutic strategy and molecular target for GC by modifying Mcl-1 pre-mRNA alternative splicing.

## RESULTS

### No significant change in Mcl-1 expression in GC tissues was observed

To investigate the characteristics of Mcl-1 distribution in GC, data on Mcl-1 expression in GC were collected from the Cancer Genome Atlas (TCGA) database and summarized. Mcl-1 expression showed a statistically non-significant increasing trend related to poor survival and higher tumor grades and cancer stages (Supplementary Figure 1). These results indicated that the Mcl-1 expression, without distinguishing Mcl-1L and Mcl-1S isoform expression, presented no substantial changes in GC tissues and no obvious association with GC development.

### Mcl-1L expression was increased while Mcl-1S expression was decreased in GC

Given that the isoforms of Mcl-1L is pro-apoptotic and the S form anti-apoptotic, respectively, we examined Mcl-1L and Mcl-1S expression in GC. Descriptive statistics of the enrolled patients, 59 with gastric adenocarcinoma and 31 with gastritis, are summarized in Supplementary Table 1. All GC patients showed similar Mcl-1S/Mcl-1L mRNA levels with respect to age (p = 0.597), sex (p = 0.927), and pathological grades (p = 0.334), and lower ratio was positively correlated with TNM staging (p <0.001). This result showed that Mcl-1L and Mcl-1S expression in GC was independent of age, sex, and pathological grades, but was related to GC progression.

**Figure 1:**
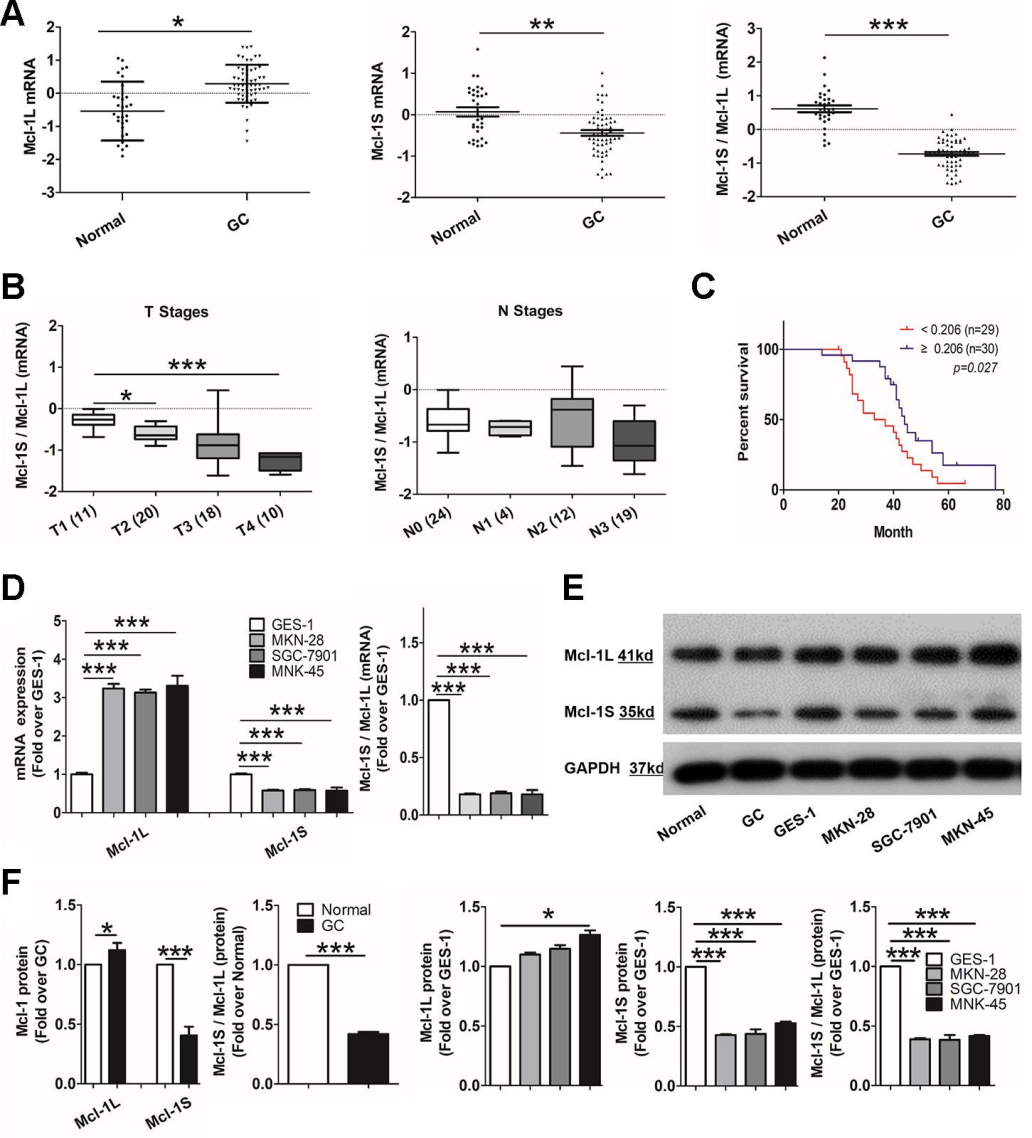
**Up-regulated myeloid cell leukemia (Mcl)-1L and down-regulated Mcl-1S expression is correlated with tumor proliferation and poor survival in human gastric cancer (GC).** (**A**) Comparison of Mcl-1L and Mcl-1S messenger RNA (mRNA) expression between GC and normal tissues was performed. Dot plots represent Mcl-1L and Mcl-1S mRNA expression levels and Mcl-1S/Mcl-1L ratios in 59 GC samples and 31 healthy tissues. Mcl-1L and Mcl-1S expression was normalized to glyceraldehyde 3-phosphate dehydrogenase expression. Data are presented after logarithmic transformation as the mean ± standard deviation (SD). *p <0.05, **p <0.01, ***p <0.001, versus the control. (**B**) The Mcl-1S/Mcl-1L mRNA ratios in cells in different T or N stages of the TNM staging system are presented. Data are shown after logarithmic transformation. (**C**) Kaplan–Meier survival curves for 59 individuals grouped based on the median value of Mcl-1S/Mcl-1L are shown. (**D**) Increased Mcl-1L and decreased Mcl-1S mRNA expression levels and Mcl-1S/Mcl-1L values in GC cell lines, compared with in the GES-1 cell line, are shown. mRNA expression was normalized by the 2^-ΔΔCt^ method. Data are shown as the means ± SD. (**E**) Western blot showing decreased Mcl-1S protein levels and Mcl-1S/Mcl-1L in GC tissues and cell lines is shown. (**F**) Increased Mcl-1L and decreased Mcl-1S protein expression levels and Mcl-1S/Mcl-1L values in GC cell lines, compared with those in the GES-1 cell line, are shown. Data are shown as the means ± SD.

Next, to confirm the role of Mcl-1L and Mcl-1S in human GC, a series of histological and molecular analyses were performed. Quantitative reverse transcription-polymerase chain reaction (RT-qPCR) results showed that Mcl-1L mRNA expression was significantly higher (p <0.05) and Mcl-1S mRNA expression was lower (p <0.01) in tumor specimens than in normal gastric mucosal tissues. Consistent with this difference, the Mcl-1S/Mcl-1L mRNA ratio was significantly decreased (p <0.001) in GC (Figure 1A). Moreover, this decrease was accompanied with progression of T stages, instead of N stages (Figure 1B). This result indicated that Mcl-1L over-expression and/or Mcl-1S under-expression was closely related to tumor size increase but not to lymph node metastasis [31]. Only one patient in the M stage developed distant metastases and showed invalid statistics. Kaplan–Meier survival analysis showed that the overall survival of patients with lower Mcl-1S/Mcl-1L levels was decreased compared with that of patients with higher levels (Figure 1C). These results suggested that high Mcl-1L and low Mcl-1S expression levels in GC tissue were correlated with tumor proliferation and prognosis.

Subsequently, we validated Mcl-1L and Mcl-1S expression using differently differentiated GC cell lines, including MKN-28 (well-differentiated), SGC-7901 (moderately-differentiated), and MKN-45 (poorly-differentiated). As shown in Figure 1D, higher Mcl-1L and lower Mcl-1S mRNA expression levels were observed in all three GC cell lines than in human gastric epithelial cells (GES-1 cells). Additionally, compared with GES-1 cells, GC cell lines showed significantly reduced Mcl-1S/Mcl-1L ratio. However, there was no difference in the Mcl-1L and Mcl-1S mRNA levels among the GC cell lines, demonstrating that up-regulated Mcl-1L and down-regulated Mcl-1S expression patterns, similar to those in GC tissues, were observed in the GC cell lines. However, they were not correlated with GC cell differentiation.

Consistent with this difference at the mRNA level, the Mcl-1 protein expression pattern was characterized by markedly higher Mcl-1L levels, lower Mcl-1S levels, and a relatively similar Mcl-1S/Mcl-1L ratio in both GC tissues and cell lines, compared with those in normal tissues and cells (Figure 1E and 1F). These results further confirmed the up-regulated Mcl-1L and down-regulated Mcl-1S expression in GC and the reversed pattern in normal tissues and cells.

Taken together, these findings suggested prominent Mcl-1L and reduced Mcl-1S expression in GC tissues and cell lines. Moreover, the lower Mcl-1S/Mcl-1L ratio contributed to gastric tumor proliferation and poor prognosis.

### Modification of Mcl-1 pre-mRNA alternative splicing using steric-blocking oligonucleotides (SBOs) in GC cell lines

**Figure 2:**
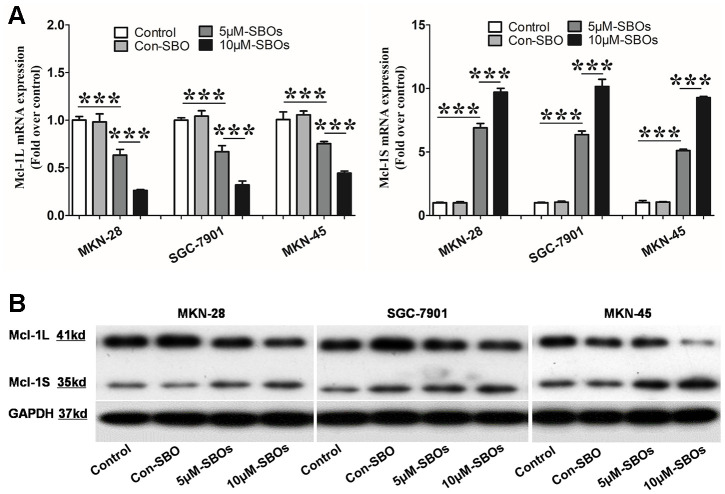
**The myeloid cell leukemia (Mcl)-1 splicing pattern shifts efficiently from Mcl-1L to Mcl-1S after delivery of the steric-blocking oligonucleotides (SBOs) into the gastric cancer (GC) cell lines.** (**A**) Mcl-1L and Mcl-1S messenger RNA (mRNA) expression levels in GC cell lines after treatment with SBOs at different dosages are shown. Data of at least three independent experiments are shown as the means ± standard deviation. (**B**) Western blot showing the Mcl-1L and Mcl-1S protein levels in GC cell lines treated with phosphate-buffered saline or SBOs at the indicated concentrations is presented. This experiment was repeated thrice.

Given the effects of the Mcl-1 isoforms on apoptosis, we speculated that blocking Mcl-1L expression and inducing Mcl-1S expression would cause the cancer cells to switch to a pro-apoptotic state and restrain tumor progression. Therefore, we transfected GC cell lines with fluorescein-tagged Mcl-1-specific SBOs to shift the Mcl-1 pre-mRNA splicing pattern from Mcl-1L to Mcl-1S. Fluorescein-tagged SBOs were delivered into the GC cell nuclei. They bound to complementary fragments with different transfection efficiencies depending on the SBO dosage (Supplemental Figure 2). After 48 h of transfection of 5 or 10 μM SBOs, Mcl-1L mRNA expression was down-regulated and Mcl-1S mRNA expression was up-regulated in a dose-dependent manner in the three GC cell lines (Figure 2A). Similar to the quantitative change in the mRNA levels, a decrease in the Mcl-1L protein expression level and an increase in the Mcl-1S protein expression level were observed in SBO-treated GC cell lines (Figure 2B). These results demonstrated that the Mcl-1-specific SBOs shifted the Mcl-1 splicing pattern from Mcl-1L to Mcl-1S efficiently, resulting in decreased anti-apoptotic Mcl-1L and increased pro-apoptotic Mcl-1S expression levels.

### Effects of the shift in Mcl-1 pre-mRNA splicing from Mcl-1L to Mcl-1S on regulation of apoptosis *in vitro*

**Figure 3:**
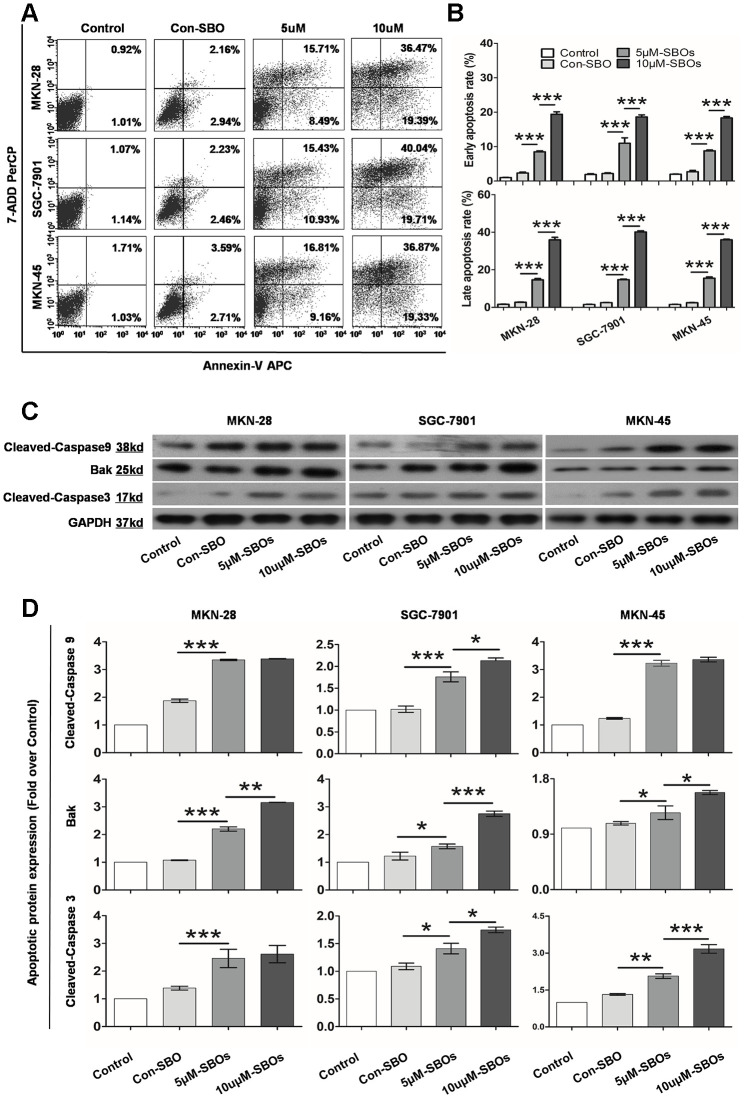
**The shift in the myeloid cell leukemia (Mcl)-1 splicing pattern from Mcl-1L to Mcl-1S promotes apoptosis of different gastric cancer (GC) cell lines.** (**A**) Flow cytometry showing the apoptosis rates of the GC cell lines treated with 5 and 10 μM steric-blocking oligonucleotides (SBOs) is shown. Early and late apoptotic cells are shown in the right lower and upper quadrants, respectively. (**B**) Pair-wise comparison of early and late apoptosis rates of SBO-treated GC cell lines is shown. Data are shown as the means ± standard deviation (SD). (**C**) Activated apoptin expression in GC cell lines treated with SBOs at the indicated dosages was detected by western blotting. (**D**) Activated apoptin expression was summarized as an integrated density value. Data are shown as the means ± SD.

To validate whether the shift in the Mcl-1 splicing pattern from Mcl-1L to Mcl-1S enhanced GC cell apoptosis, the apoptotic cells were quantified by flow cytometry (FCM). Annexin V and 7-amino-actinomycin (7-AAD) were used to identify the early and late apoptotic cells, respectively. The results indicated that 48 h post-transfection of SBOs, the number of apoptotic cells increased in the three GC lines (Figure 3A). The apoptosis rates are summarized in Figure 3B, which shows that SBOs result in a significant dose-dependent increase in the number of both early and late apoptotic cells. Western blotting (WB) was performed to evaluate apoptosis by detecting the key markers of apoptosis (Bak, cleaved caspase 9, and cleaved caspase 3) (Figure 3C). The integrated density values indicated that the expression of apoptotic factors in SBO-treated GC cell lines showed statistically significant acceleration with a clear dose-response relationship (Figure 3D). Collectively, these data verified that modification of the Mcl-1 splicing pattern from Mcl-1L to Mcl-1S using SBOs strikingly stimulated GC cell apoptosis.

### Modification of Mcl-1 pre-mRNA alternative splicing from Mcl-1L to Mcl-1S *in vivo*

**Figure 4:**
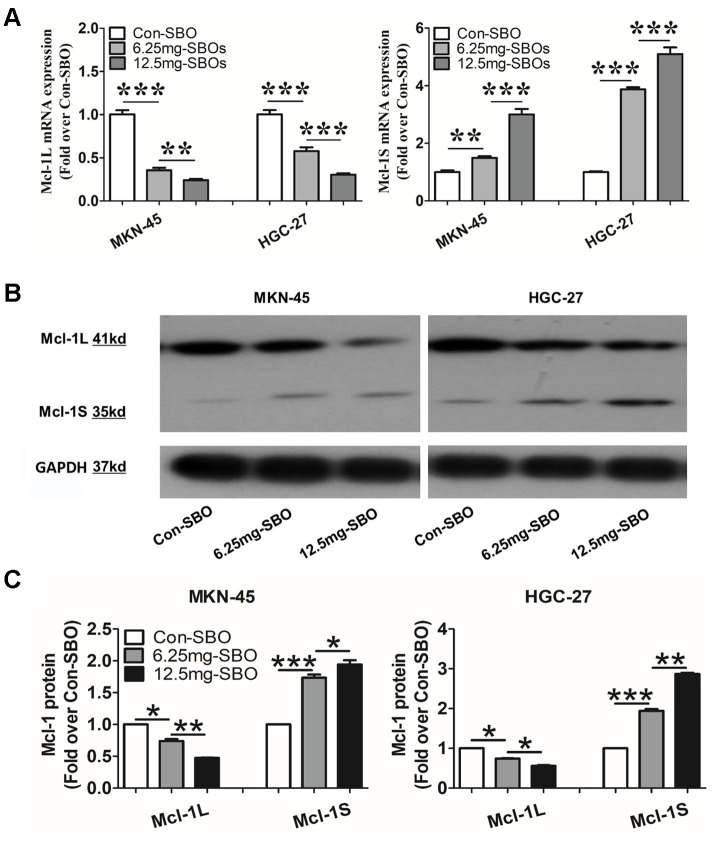
**The myeloid cell leukemia (Mcl)-1 splicing shifts from Mcl-1L to Mcl-1S after injection of the vivo-morpholino-modified steric-blocking oligonucleotides (SBOs) in the mouse xenograft models of MKN-45 and HGC-27 cells.** (**A**) Mcl-1L and Mcl-1S messenger RNA (mRNA) expression in the MKN-45 and HGC-27 xenograft models treated with vivo-morpholino-modified SBOs at different dosages is presented. Data are shown as the means ± standard deviation (SD). **p <0.01, ***p <0.001, versus the control. (**B**) Mcl-1L and Mcl-1S protein expression in the xenograft models after treatment with SBOs at indicated doses is shown. (**C**) Decreased Mcl-1L and increased Mcl-1S protein expression levels and Mcl-1S/Mcl-1L values after SBO treatment are presented. Data are shown as the means ± SD. *p <0.05, **p <0.01, ***p <0.001 versus the control.

The tumorigenicity assay in male severe combined immune-deficient mice was performed to investigate whether modification of Mcl-1 splicing via SBO treatment affected tumor cell apoptosis and growth *in vivo*. Mcl-1S mRNA was up-regulated while Mcl-1L mRNA was prominently down-regulated, depending on the SBO dosage in the mouse xenograft models of both MKN-45 and HGC-27 cells (Figure 4A). As expected, Mcl-1L and Mcl-1S protein levels were altered in parallel with their mRNA expressions (Figures 4B and 4C). Mcl-1S/Mcl-1L ratio was also significantly increased in the SBO-treated MKN-45 cells (Figure 4C). These results indicated that Mcl-1-specific SBOs succeeded in blocking Mcl-1L expression and inducing Mcl-1S expression in mice.

### The role of altered Mcl-1 pre-mRNA splicing in promoting apoptosis and suppressing GC *in vivo*

**Figure 5:**
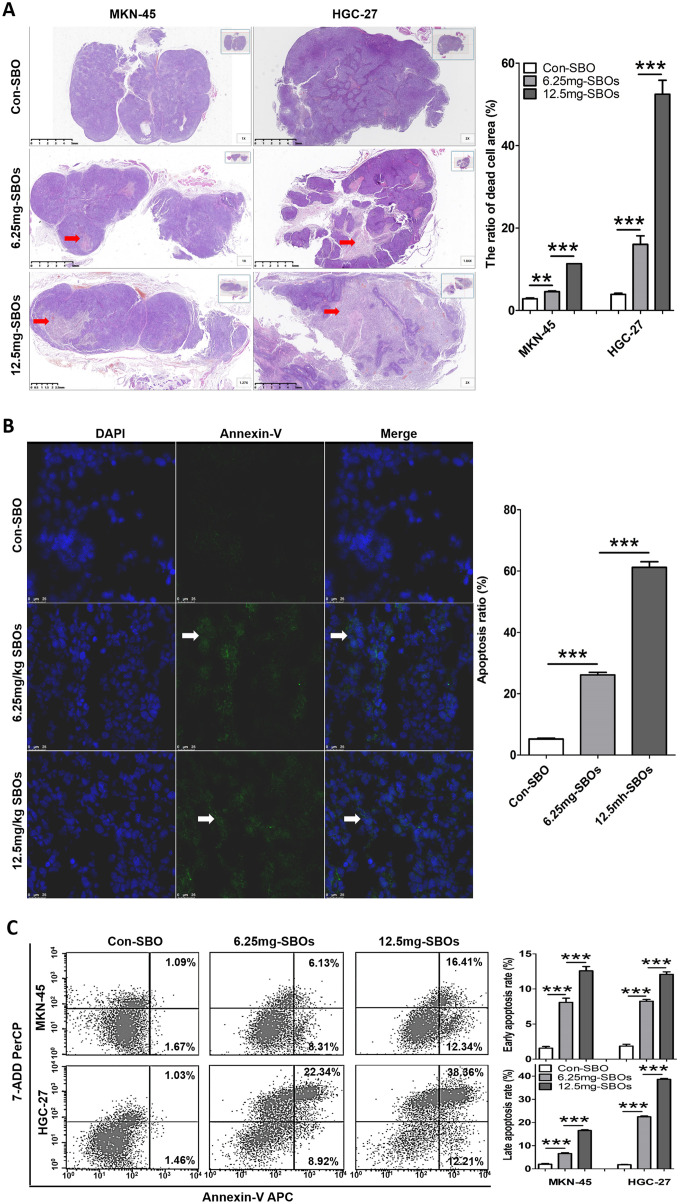
**Altered myeloid cell leukemia (Mcl)-1 splicing from Mcl-1L to Mcl-1S promotes apoptosis *in vivo*.** (**A**) Hematoxylin and eosin staining of tumor sections of the xenograft models is shown. The red arrows indicate dead cells. The dead cell area/overall tumor area increased in a dose-dependent manner after steric-blocking oligonucleotide (SBO) treatment. (**B**) Immunofluorescence with annexin V staining (the white arrows indicate green fluorescence) showing apoptotic cells in the tumor sections of the HGC-27 xenograft models is presented. The results of the between-group and repeated-measure analyses are shown as the means ± standard deviation (SD). (**C**) Flow cytometry showing the apoptosis rates of tumor cells treated with SBOs at indicated dosages is presented. Between-group comparison results are shown as the means ± SD.

To validate whether Mcl-1 pre-mRNA alternative splicing using SBOs could induce tumor cell apoptosis *in vivo*, apoptotic cells were detected in the xenograft models. In the mouse xenograft models of both MKN-45 and HGC-27 cells, hematoxylin and eosin (HE) staining revealed a dose-dependent increase in the dead cell area of the SBO-treated tumor tissue, compared with that of the control (Figure 5A). Immunofluorescence assay of the HGC-27 xenograft tumors demonstrated that SBO treatment resulted in a dose-dependent increase in the number of apoptotic cells (annexin V-positive) (Figure 5B); identical results were observed in the MKN-45 xenograft models (not presented in current study). Correspondingly, quantitation by FCM showed that treatment with 0, 6.25, and 12.5 mg/kg SBOs led to apoptosis rates of 2.76, 14.44, and 28.75% in the MKN-45 xenograft models and 2.49, 31.26, and 50.57% in the HGC-27 xenograft models, respectively. The results showed that the early and late apoptosis rates increased in a statistically significant dose-dependent manner (Figure 5C). Additionally, after Mcl-1-specific SBO treatment for 3 d, Bak, activated caspase 9, and caspase 3 expression levels in the tumor tissues were markedly elevated in a dose-dependent manner (Supplementary Figure 3). These results indicated that the shift in Mcl-1 splicing from Mcl-1L to Mcl-1S induced significant cell apoptosis in gastric xenograft tumors.

**Figure 6:**
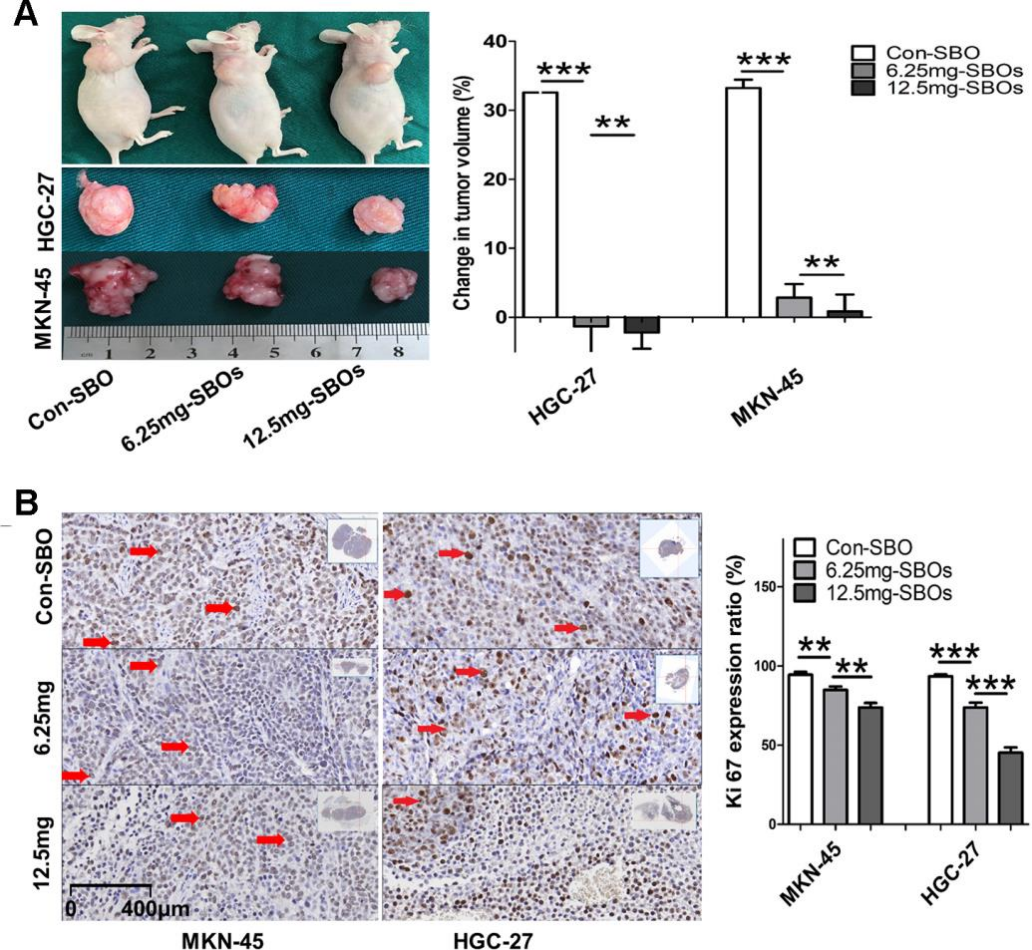
**Shifted myeloid cell leukemia (Mcl)-1 splicing from Mcl-1L to Mcl-1S suppresses gastric cancer (GC) proliferation *in vivo*.** (**A**) Change in tumor volume ([end-point volume – initial volume]/initial volume) after treatment with steric-blocking oligonucleotides (SBOs) at indicated dosages is presented. The results of the between-group analyses are shown as the means ± standard deviation. (**B**) Immunohistochemical staining showing Ki-67 expression levels (red arrows) in tumor sections of the xenograft models, exhibiting changes in tumor viability and proliferation, after treatment with SBOs at indicated dosages is shown.

Next, we examined the effects of Mcl-1-specific SBO treatment, which caused a shift in Mcl-1 splicing, on tumors *in vivo*. The volumes of the SBO-treated tumors remained stable, while those of the Con-SBO-treated tumors experienced growth rates of over 30%. In the mouse HGC-27 xenograft models, the volumes of the tumors treated with 6.25 and 12.5 mg/kg SBOs reduced by 1.32% and 2.21%, respectively, while in the mouse MKN-45 xenograft models, the volumes increased by 2.85% and 0.85%, respectively (Figure 6A). Additionally, immunohistochemical detection of Ki-67, performed to evaluate tumor viability and proliferation, demonstrated a significant dose-dependent decrease in the Ki-67 expression rate in the SBO-treatment groups (Figure 6B). These results indicated that Mcl-1S over-expression and Mcl-1L under-expression, regulated by Mcl-1-specific SBO treatment, inhibited tumor proliferation *in vivo* in this model.

## DISCUSSION

GC is the second leading cause of cancer mortality in the world [32]. Surgery and chemoradiotherapy remain the current major therapeutic options for GC, but outcomes are still unfavorable [33, 34]. It has been well established that alternative splicing contributes to pathological alterations that promote cancer initiation and/or maintenance [35]. More importantly, new trends in cancer research have shown that alternative splicing has clinical potential in cancer therapy [15, 36]. Our study indicated that the anti-apoptotic Mcl-1L and pro-apoptotic Mcl-1S proteins, the major Mcl-1 splicing isoforms, were up-regulated and down-regulated, respectively, in GC. Moreover, increased Mcl-1L and decreased Mcl-1S levels contributed to gastric tumor proliferation and poor prognosis. Additionally, this study confirmed that modification of Mcl-1 splicing from Mcl-1L to Mcl-1S using SBOs could markedly enhance apoptosis and inhibit proliferation in GC. The present work supplemented an innovative direction towards tumor treatment involving targeting apoptotic proteins through the manipulation of alternative splicing.

This is the first study to illustrate the roles of Mcl-1L and Mcl-1S in GC systematically and verify the therapeutic effects of the modulated Mcl-1 splicing pattern in this disease; up-regulated Mcl-l L and down-regulated Mcl-1S expression in AGS cells, a human gastric adenocarcinoma epithelial cell line, has been previously demonstrated [29]. In this study, Mcl-1L and Mcl-1S expression was detected in three GC cell lines, and the results validated the previous findings. Furthermore, RT-qPCR and WB were performed for GC tissues, confirming that Mcl-1L over-expression and Mcl-1S under-expression induced tumor proliferation and shorter survival for the first time. Previously, the effects of a shift in the splicing pattern from Mcl-1L to Mcl-1S on enhancing apoptosis were solely reported based on *in vitro* analysis [29, 30]; however, the current study verified the function of the splicing pattern in regulating apoptosis both *in vitro* and *in vivo*. This result further strengthened the feasibility of targeting alternative splicing of apoptotic proteins to facilitate tumor cell death, and consequently suppress GC. Additionally, we found increased effects of SBOs in HGC-27 cells, compared to those in MKN-45 cells. Previous studies suggested that alternative splicing was regulated according to the cell type, developmental stage, and disease state [37–39]. Hence, we assumed that different cell lines might respond differently to the splicing pattern.

It has been noted that there are three alternative splicing variants of the human Mcl-1 gene, including Mcl-1L, Mcl-1S, and Mcl-1ES. Even though Mcl-1ES has been identified as a minor RT-qPCR product in several cancers and immortalized cell lines, no endogenous protein has been detected for it. Moreover, Mcl-1ES protein functions have been studied only in over-expression conditions, revealing that Mcl-1ES dimerizes with Mcl-1L and induces mitochondrial cell death [26, 40, 41]. In this study, we demonstrated predominant Mcl-1L expression and low Mcl-1S levels; however, Mcl-1ES expression was undetectable in both GC tissues and cell lines. It was possible that Mcl-1ES was not detected by RT-qPCR due to the weak expression level and short half-life of Mcl-1ES pre-mRNA. Thus, it remained unclear whether Mcl-1ES was expressed in GC. However, it is well established that Mcl-1L and Mcl-1S are the major products of the Mcl-1 gene and key factors involved in regulating apoptosis [26, 42]. Therefore, as demonstrated in this study, the modification of Mcl-1 pre-mRNA alternative splicing from Mcl-1L to Mcl-1S might suffice in triggering apoptosis and anticancer effects.

SBOs have been successfully employed to manipulate the pre-mRNA splicing pattern [43, 44]. Due to the highly degenerate nature of pre-mRNA, SBO treatment does not result in the general inhibition of splicing, but instead in the shift of the spliceosome to another splice site, consequently blocking an alternative splicing pattern and promoting the target splicing pattern [45, 46]. Additionally, SBOs with the deoxyribose sugar moieties replaced by morpholino oligos, which are not recognized by enzymes, are completely resistant to nucleases [47]. Furthermore, in this study, the Endo-Porter transfection system replaced the conventional transfection system to improve the transfection efficiency *in vitro*. Endo-Porter delivers SBOs into the cytosol of the cells by an endocytosis-mediated process that avoids damage to the cell plasma membrane and the loss of vital cell contents and associated toxicity [48]. Vivo-morpholinos are exon-skipping reagents of choice for *in vivo* experiments because they show outstanding results due to their attractive profile of stability, low toxicity, and good cell penetration. These are assembled by coupling the vivo-delivery group to a morpholino while the oligo is still bound to its synthesis resin, allowing excellent purification on washing the solid-phase resin [49]. These advanced technologies helped guarantee the fidelity of our study.

The molecular mechanisms underlying unbalanced Mcl-1 mRNA splicing, which lead to up-regulated Mcl-1L and down-regulated Mcl-1S in GC, were not investigated in this study. Current research indicates that serine arginine-rich splicing factor (SRSF) and RNA-binding motif protein 4 (RBM4) are pivotal splicing factors involved in the regulation of Mcl-1 alternative splicing. SRSF1 and SF3B1 favored Mcl-1L formation, while RBM4 and SRSF2 promoted the skipping of exon 2 in Mcl-1 pre-mRNA and contributed to Mcl-1S expression [50, 51]. Coincidentally, recent studies suggested that SRSF1 in GC tissues was up-regulated and associated with poor outcome [52]. It induced apoptosis in the AGS and MKN-28 human GC cells *in vitro* [53]. Je et al. (2013) found that SRSF2 expression reduced (up to 7-fold) in gastric tumors [54], while Yong et al. (2016) reported that both RBM4 protein and mRNA expression levels were significantly lower in GC tissues than in the adjacent non-cancerous tissues [55]. These results suggested that the abnormal expression of these splicing factors in GC might disturb Mcl-1 alternative splicing and consequently up-regulate Mcl-1L expression and down-regulate Mcl-1S expression, resulting in apoptosis resistance in GC. Hence, further research is necessary to elucidate the molecular mechanisms underlying aberrant Mcl-1 splicing by targeting these splicing factors in GC and understand the potential role of Mcl-1 mRNA splicing in GC therapy.

However, the current study has two limitations. Firstly, Mcl-1L and Mcl-1S expression in the normal tissues adjacent to the GC tissues was absent. The effects of the docetaxel and cisplatin combination treatment on Mcl-1L and Mcl-1S expression were also absent. Additionally, we found that SBO treatment promoted normal GES-1 cell apoptosis, but the apoptosis rate was obviously lower in the GES-1 cells than in the GC cells (Supplementary Figure 4). However, the GC xenograft mice showed no obvious damage to organs, including the liver, kidneys, lungs, and pancreas, after SBO treatment (Supplementary Figure 5). Meanwhile, no significant change in the body weight of the GC xenograft mice was observed after SBO treatment (Supplementary Figure 6). Hence, SBO toxicity requires further investigation.

The unbalanced anti- and pro-apoptotic protein expression, which resulted from aberrant splicing of apoptosis-related genes, is a major feature of many cancers. Thus, modulating isoform expression of apoptotic proteins by targeting alternative splicing is a promising strategy to facilitate tumor cell death and suppress cancer. This study demonstrated that modification of Mcl-1 mRNA splicing from Mcl-1L to Mcl-1S facilitated GC cell apoptosis in both a cell-culture system and mouse models, representing an exploratory work on targeting alternative splicing to stimulate pro-apoptotic factors and inhibit anti-apoptotic proteins for GC therapy.

## MATERIALS AND METHODS

### Gene set enrichment analysis (GSEA)

Mcl-1 mRNA data used for GSEA are accessible from TCGA database (https://tcga-data.nci.nih.gov/docs/publications/tcga/). The correlations among Mcl-1 mRNA level, tumor grade, and cancer stage were analyzed using the software Gene Expression Profiling Interactive Analysis (GEPIA) (http://gepia.cancer-pku.cn/). The median Mcl-1 expression level was used as the parameter to divide the high and low groups of clinical GC specimens. Statistical significance (false discovery rate) was set at 0.05.

### Patients and clinical data

Fifty-nine gastric adenocarcinoma and thirty-one gastritis patients were recruited for this study after admission at the Gansu Provincial Hospital. The diagnosis, grading, and staging of gastric adenocarcinoma were established according to the NCCN Clinical Practice Guidelines in Oncology: Gastric Cancer (Version 2.2018). Kaplan–Meier survival curves for GC patients with low and high Mcl-1S/Mcl-1L mRNA ratios (grouped according to the median value of the Mcl-1S/Mcl-1L ratio) were generated by retrospective follow-up. The protocol of this study followed the ethical guidelines of the 1975 Declaration of Helsinki, and the study was approved by the Ethics Review Committee of Gansu Provincial Hospital. All participants provided their written informed consent to participate in this study.

### Cell culture

Authenticated and differently differentiated GC cell lines (MKN-28, SGC-7901, and MKN-45) were obtained from Beijing Fenghui Biotechnology Co. Ltd. (China). The cells were incubated at 37°C in 5% CO_2_ for 4–6 h and then immediately cultured in RPMI-1640 medium supplemented with 10% fetal bovine serum (9:1) and penicillin/streptomycin (100 U/mL) (Invitrogen Corporation, Carlsbad, CA, USA) at 37°C in 5% CO_2_. Cells in the logarithmic growth stage were digested, seeded into 6-well plates (4×10^5^ cells/mL in each well), incubated at 37°C for 24 h, digested with pancreatin, and collected for further research.

### Modification of Mcl-1 pre-mRNA alternative splicing

SBOs were used to cause down- and up-regulation of Mcl-1L and Mcl-1S expression, respectively. The SBOs were synthesized, and the Endo-Porter delivery system was purchased from Gene Tools (Philomath, OR, USA). A pair of SBOs was designed such that they could bind to the 3'-acceptor and 5'-donor-splicing site of exon 2 of Mcl-1 pre-mRNA and splice out exon 2, thereby shifting the splicing pattern from Mcl-1L to Mcl-1S. The SBO sequences were 5'-CGAAGCATGCCTGAGAAAGAAAAGC-3' and 5'-AAGGCAAACTTACCCAGCCTCTTTG-3'. The SBOs blocked the 5'-donor and 3'-acceptor sites of exon 2 to skip exon 2, thereby shifting the splicing pattern from Mcl-1L to Mcl-1S mRNA. A non-targeting oligonucleotide sequence was cloned as the control: 5'-CCTCTTACCTCAGTTACAATTTATA-3' (Con-SBO). The Endo-Porter delivery system was used to optimize the conditions, according to the manufacturer’s instructions. GC cell lines were treated with 5 and 10 μM Mcl-1-specific SBOs for a verified optimum period of 48 h [29]. Localization and transfection efficiency of fluorescein-tagged SBOs were defined by laser confocal microscopy. RT-qPCR and WB analyses were performed to confirm Mcl-1L and Mcl-1S expression. FCM was used for counting the apoptotic cells.

### Xenotransplantation and tumor analysis

All animal experiments were performed according to the Gene Tools and ARRIVE guidelines [56]. The protocol of this study was approved by the Ethics Review Committee of Gansu University of Chinese Medicine. Briefly, MKN-45 or HGC-27 cells (1×10^7^ cells) in serum-free medium (100 μL) were implanted into the subcutaneous tissue of the antedorsal walls of immunodeficient mice (4–5-week-old males, one tumor per mouse, n = six mice per group). Mice were grouped after tumor formation and then injected with vivo-morpholino-modified Mcl-1-specific SBOs or a non-targeting vivo-morpholino-modified oligonucleotide (as the control) (Gene Tools, LLC, Pacific Grove, CA) by local multipoint administration. Dosing (1.25 or 6.25 mg/kg SBOs) was repeated daily for 3 days, and the mice were sacrificed on day 4. The tumor volumes were measured with calipers and calculated using the following equations: volume = length × (width)^2^ × 0.5; change in tumor volume = (terminal volume – initial volume)/initial volume. Each tumor sample was snap-frozen in liquid nitrogen or fixed immediately in paraformaldehyde and then processed for further blind histological analysis.

### RT-qPCR

Total RNA was extracted using TRIzol reagent, and cDNA was synthesized using the RNeasy mini kit (Qiagen, Germany). cDNA was used for RT-qPCR using Taqman universal PCR master mix (ABI, 4444556, USA). Mcl-1L and Mcl-1S mRNA was detected with Mcl-1 isoform-specific gene expression probes (ABI, Mcl-1L: Hs00172036_m1; Mcl-1S: Hs00766187_m1) and normalized to glyceraldehyde 3-phosphate dehydrogenase (GAPDH) mRNA (ABI, Hs99999905_m1). RT-qPCR reactions were performed at 95°C for 10 min with 40 cycles at 95°C for 15 s, 60°C for 30 s, and 70°C for 30 s. Mcl-1L and Mcl-1S expression was analyzed using the comparative threshold cycle (Ct) method of relative quantification.

### WB

Proteins were extracted from tumor tissues and purified using total protein extraction kits (KeyGEN, China). Protein concentration was detected and normalized by the bicinchoninic acid method. Denatured samples were resolved on 12% (w/v) SDS-PAGE gels and transferred onto PVDF membranes (Millipore, USA) by the wet transfer-coating finishing method. The PVDF membrane was immersed in 5% skimmed milk in tris-buffered saline at room temperature for 1 h and then incubated overnight with rabbit anti-human Mcl-1 antibody (1:1,000) (Bio-Rad, AHP998, USA), which can recognize both the Mcl-1L (41 kDa) and Mcl-1S (35 kDa) proteins, at 4°C. Rabbit polyclonal antibody against GAPDH (Bio-Rad, USA) was loaded as the control; the secondary antibody was horseradish peroxidase-conjugated IgG (1:5,000) (Bio-Rad, USA). Densitometry analysis was performed using ImageJ software (Bio-Rad, USA).

### Apoptosis detection by FCM

GC and transplanted tumor cell apoptosis was detected using the Annexin V-APC/7-AAD apoptosis detection kit (Multisciences Biotech, China). Cells (10,000 cells per sample) were re-suspended in a binding buffer, stained with annexin V-APC and 7-AAD for 15 min in the dark, and diluted with the binding buffer to obtain a final volume of 500 mL. Data acquisition and analysis were performed using a flow cytometer (FACS Caliber, BD, USA).

### Immunofluorescence

The paraffin sections of the tumor samples were stained with rabbit anti-annexin V antibody, goat anti-rabbit IgG H&L (Alexa Fluor® 488) (Abcam, USA), and DAPI (BD, USA) to determine tumor tissue apoptosis. Images were captured using a confocal microscope (Leica Microsystems, SP8, Germany) on standard settings.

### Immunohistochemistry

Paraffin tumor sections (6 μm) were HE stained to detect dead cells and immunostained with antibodies against Ki-67 (Protein Tech Group, China) to analyze tumor proliferation. The dead cell area and Ki-67 positive rate were analyzed using KF-PRO-020 (KFBIO, China).

### Statistical analyses

Statistical analysis was performed using SPSS 18.0 software. Data of at least three independent experiments for each of the cellular and animal groups were presented as the mean ± standard deviation. The differences in the measurement data among the groups were analyzed by the homogeneity test of variances. The significance of differences among individual groups was analyzed by Student’s t-test (two-tailed). One-way ANOVA was performed for multiple group comparisons. Survival curves were plotted using the Kaplan–Meier method and compared using the log-rank test. We defined a high expression level as that above the median and a low expression level as that below the median. All statistical tests were two-sided, and P <0.05 indicated statistical significance.

## Supplementary Material

Supplementary Figures

Supplementary Table 1
