# Supplementary material for: Modification of Mcl-1 alternative splicing induces apoptosis and suppresses tumor proliferation in gastric cancer
Source: Aging (Albany NY). 2020 Oct 14;12(19):19293–315. doi: 10.18632/aging.103766 (PMC7732305; doi:10.18632/aging.103766)
Supplement: Supplementary Figures [file aging-12-103766-s001..pdf]

SUPPLEMENTARY FIGURES

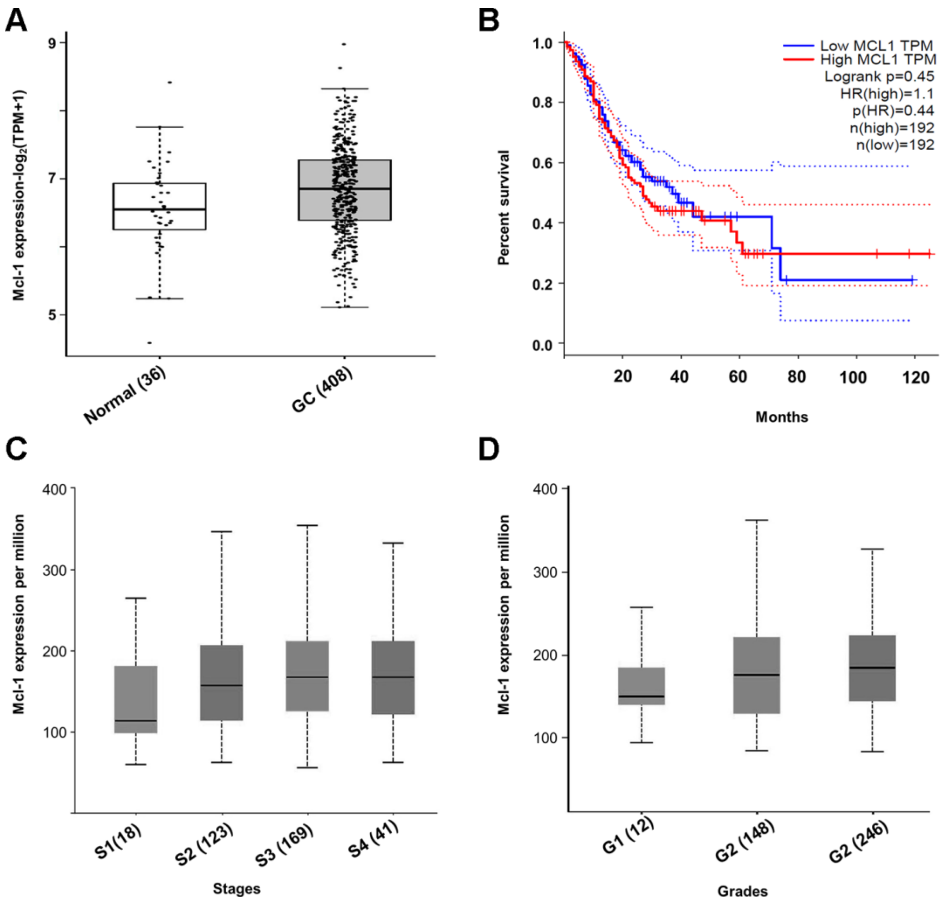

Supplementary Figure 1. Myeloid cell leukemia (Mcl)-1 shows an increasing trend in gastric cancer (GC) tissues, and is associated with GC development. (**A**) Mcl-1 expression levels in GC (n = 408) and normal gastric (n = 36) tissues, according to the data from the Cancer Genome Atlas database, are shown (P >0.05). (**B**) Kaplan–Meier analysis of overall survival based on Mcl-1 expression in GC is shown (P = 0.44). (**C**) Intra-group comparison of Mcl-1 expression in different GC stages is shown (P >0.05). (**D**) Mcl-1 expression in GC based on tumor grades is shown (P >0.05).

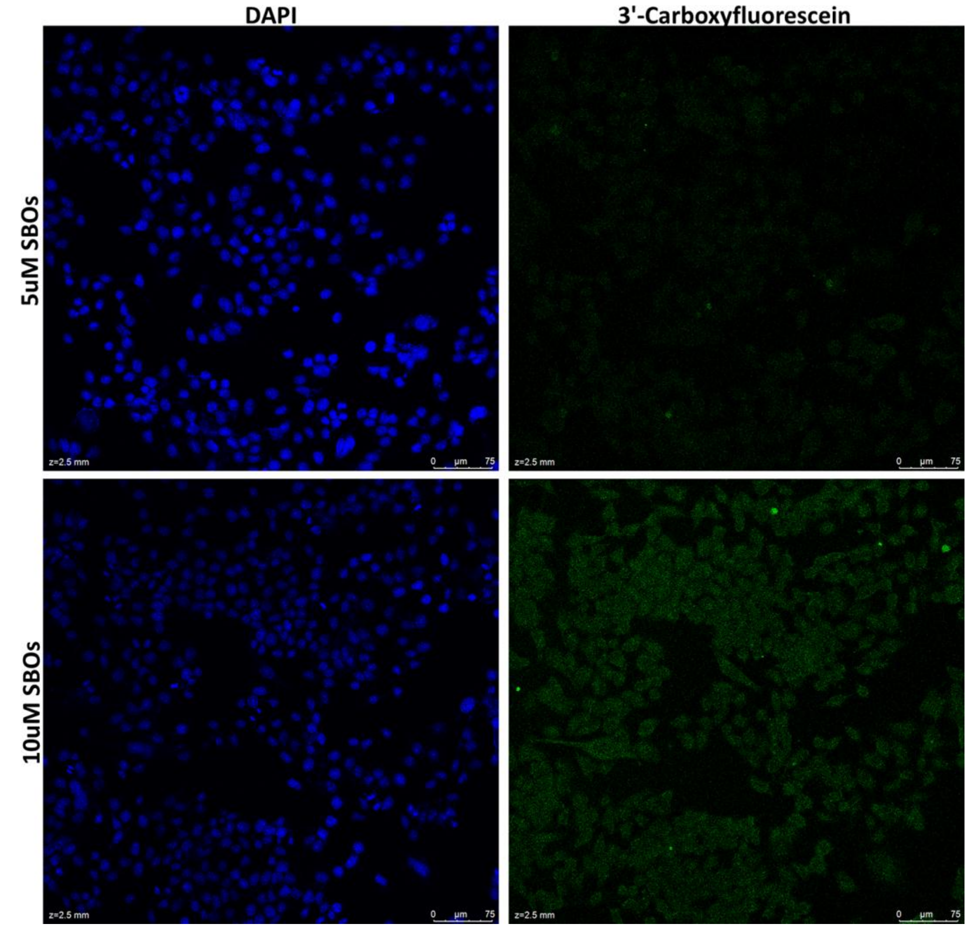

Supplementary Figure 2. Localization and delivery efficiencies of steric-blocking oligonucleotides (SBOs) are shown. Pictures were collected by laser confocal microscopy. Green fluorescence signal represents the binding of the delivered SBOs to the target RNA, and it increased with the increase in the dose of Endo-Porter and SBOs.

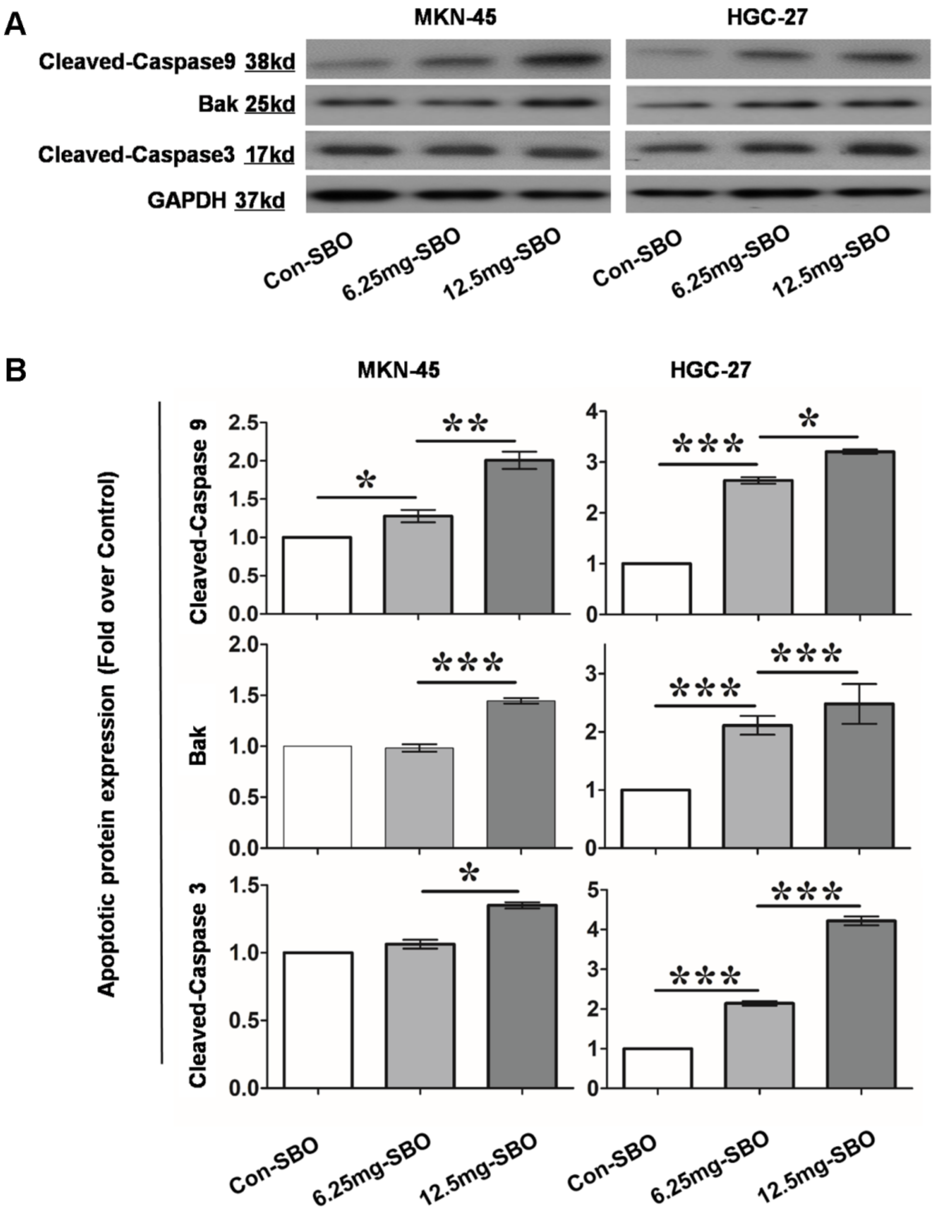

Supplementary Figure 3. Dose-dependent increases in Bax, activated caspase 9, and caspase 3 in tumor tissues treated with steric-blocking oligonucleotides (SBOs) at the indicated dosages are observed. (**A**) Activated apoptin expression detected by western blotting is shown. (**B**) Statistical analysis of the integrated density value of activated apoptin expression is shown.

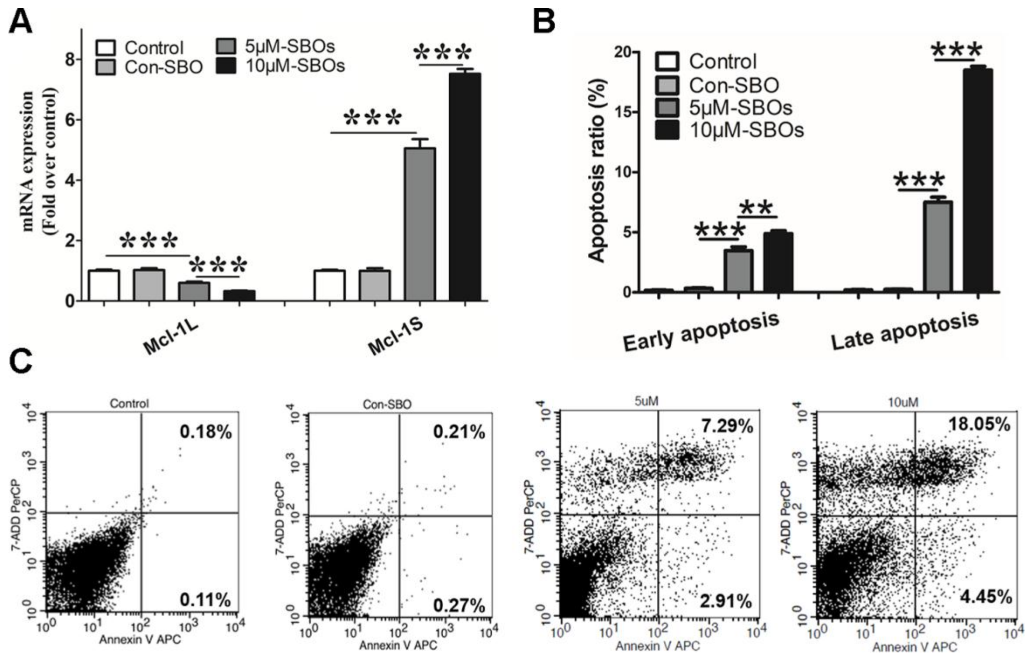

Supplementary Figure 4. Steric-blocking oligonucleotide (SBO) treatment promotes normal GES-1 cell apoptosis. (**A**) The myeloid cell leukemia (Mcl)-1L and Mcl-1S messenger RNA (mRNA) levels were measured by quantitative reverse transcription-polymerase chain reaction after SBO treatment. (**B**) Pair-wise comparison of the early and late apoptosis rates of the SBO-treated gastric cancer (GC) cell lines is shown. Data are shown as the means ± standard deviation. (**C**) Flow cytometry showing apoptosis rates of the GC cell lines treated with 5 and 10 μM SBOs is shown. Early and late apoptotic cells are shown in the right lower and upper quadrants, respectively. ***p <0.001.

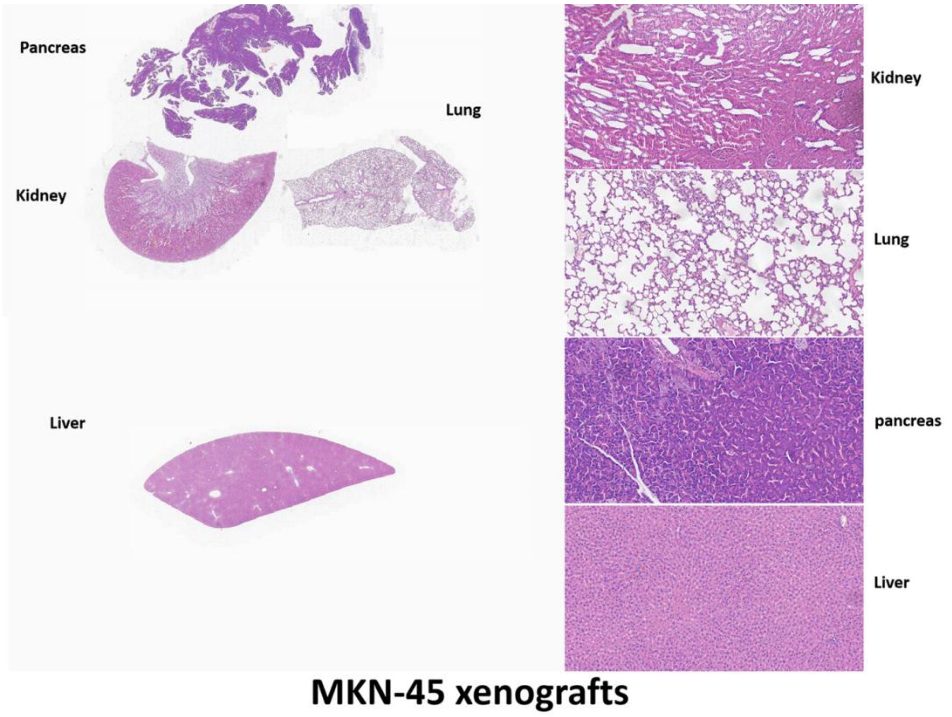

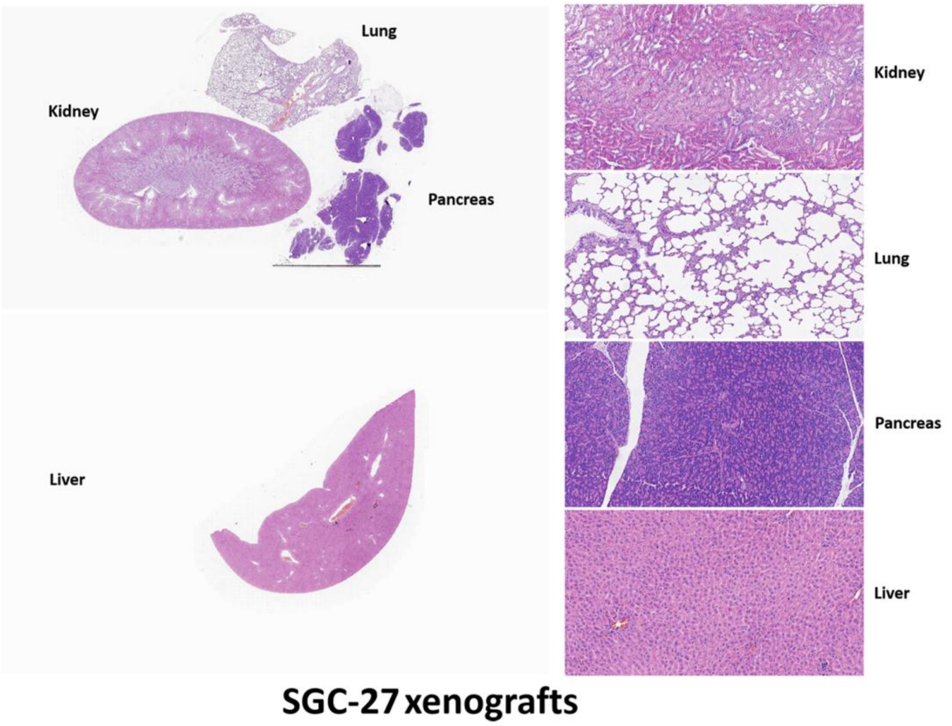

Supplementary Figure 5. Gastric cancer xenograft mice show no obvious damage to organs, including the liver, kidneys, lungs, and pancreas, after steric-blocking oligonucleotide (SBO) treatment. The SBO-treated liver, kidney, lung, and pancreas tissues were analyzed by hematoxylin and eosin staining.

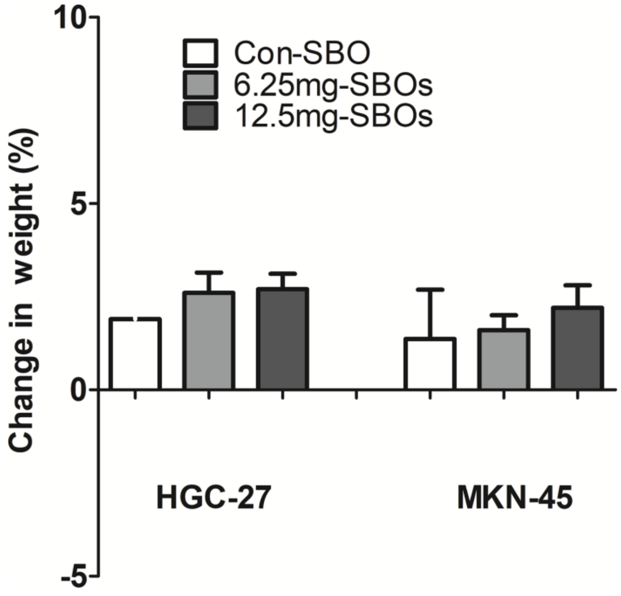

Supplementary Figure 6. No significant changes in the body weight of the gastric cancer xenograft mice after steric-blocking oligonucleotide treatment are observed.
