# Supplementary material for: Modification of Mcl-1 alternative splicing induces apoptosis and suppresses tumor proliferation in gastric cancer
Source: Aging (Albany NY). 2020 Oct 14;12(19):19293–315. doi: 10.18632/aging.103766 (PMC7732305; doi:10.18632/aging.103766)
Supplement: Supplementary Table 1 [file aging-12-103766-s002..pdf]

SUPPLEMENTARY TABLE

Supplementary Table 1. The clinical characteristics and the ratio of Mcl-1 isoform in enrolled patients with gastric adenocarcinoma.

Variables	n (%)	Mcl-1S / Mcl-1L (mRNA)	*p* value
**Age (years)**			0.597
<57 (Median)	26 (44.07%)	0.27±0.17	
≥57	33 (55.93%)	0.33±0.51	
**Sex**			0.927
Male	38 (64.41%)	0.30±0.45	
Female	21 (35.59%)	0.31±0.28	
**TNM stage (Clinical)**			<0.001
I	8 (13.56%)	0.59±0.23	
IIA	8 (13.56%)	0.20±0.03	
IIB	12 (20.34%)	0.18±0.10	
IIIA	8 (13.56%)	0.79±0.82	
IIIB	4 (6.78%)	0.06±0.02	
IIIC	18 (30.51%)	0.16±0.15	
IV	1 (1.69%)	0.21	
**Pathologic grades**			0.334
G1	16 (27.12%)	0.30±0.21	
G2	20 (33.90%)	0.21±0.22	
G3	23 (38.98%)	0.39±0.57	

Age group was distinguished based on the median age. All data meet normal distribution and independent-sample t test was used for comparisons between two groups. One-way ANOVA was performed for intergroup difference. Data are presented as the mean±SD. P<0.05 indicated statistical significance.
